# Experimental evidence for convergent evolution of maternal care heuristics in industrialized and small-scale populations

**DOI:** 10.1098/rsos.140518

**Published:** 2015-06-24

**Authors:** Geoff Kushnick, Ben Hanowell, Jun-Hong Kim, Banrida Langstieh, Vittorio Magnano, Katalin Oláh

**Affiliations:** 1 School of Archaeology and Anthropology, The Australian National University, Canberra, Australia; 2 Analytics Department, Redfin Corporation, Seattle, WA, USA; 3 Institute of Cross-Cultural Studies, Seoul National University, Seoul, South Korea; 4 Department of Anthropology, North-Eastern Hill University, Shillong, India; 5 Department of Dentistry, Universidad Europea de Valencia, Valencia, Spain; 6 Department of Cognitive Psychology, Eötvös Loránd University, Budapest, Hungary; 7 Institute of Cognitive Neuroscience, Hungarian Academy of Sciences, Budapest, Hungary

**Keywords:** behavioural ecology, maternal care, convergent evolution, vignette experiment

## Abstract

Maternal care decision rules should evolve responsiveness to factors impinging on the fitness pay-offs of care. Because the caretaking environments common in industrialized and small-scale societies vary in predictable ways, we hypothesize that heuristics guiding maternal behaviour will also differ between these two types of populations. We used a factorial vignette experiment to elicit third-party judgements about likely caretaking decisions of a hypothetical mother and her child when various fitness-relevant factors (maternal age and access to resources, and offspring age, sex and quality) were varied systematically in seven populations—three industrialized and four small-scale. Despite considerable variation in responses, we found that three of five main effects, and the two severity effects, exhibited statistically significant industrialized/ small-scale population differences. All differences could be explained as adaptive solutions to industrialized versus small-scale caretaking environments. Further, we found gradients in the relationship between the population-specific estimates and national-level socio-economic indicators, further implicating important aspects of the variation in industrialized and small-scale caretaking environments in shaping heuristics. Although there is mounting evidence for a genetic component to human maternal behaviour, there is no current evidence for interpopulation variation in candidate genes. We nonetheless suggest that heuristics guiding maternal behaviour in diverse societies emerge via convergent evolution in response to similar selective pressures.

## 1. Introduction

Maternal care behaviour should be modulated in response to factors impinging on the fitness pay-offs of that care, which include maternal, offspring and environmental factors [1–3]. Following convention, we conceptualize the benefits of care as increases in the direct fitness of current offspring, and the costs as lost ability to invest in future offspring or other components of maternal fitness [4,5]. This sort of responsiveness to fitness-relevant factors may increase maternal fitness but evolves only when the fitness benefits outweigh the costs of plasticity [6,7]. For this reason, decision rules that hinge on a subset of the fitness-relevant factors may net actors higher pay-offs than decision rules that account for all possible factors [8,9]. Theoretical and empirical work suggests that mothers may use these sorts of ‘simple heuristics’ for maternal care decision-making [3,5,10]. We predict that societies facing similar selective pressures will develop similar maternal caretaking heuristics via convergent evolution [11].

To test this idea, we used a factorial vignette experiment to compare maternal care decision-making in a sample of seven populations: three industrialized and four small-scale (see table 1, and the map in the electronic supplementary material, figure S1, and the descriptions in the electronic supplementary material, appendix S1). We asked women to judge how likely a hypothetical woman was to provide care to her child in response to systematically varying maternal and offspring characteristics, and the severity of the caretaking scenario for the mother and her child. This approach allowed us to leverage the collective knowledge of women in a given population about the heuristics used in childcare decision-making and to standardize the situations presented for response [3]. Further, the factorial experimental design is well suited for studying phenomena with multiple causal variables [12], as it allowed us to estimate the effect of all factors and their interactions without confounding [13].

**Table 1.** Study populations and national-level socio-economic indicators.

country	population(s)	TFR^a^	IMR^a^	health expenditures^b^	oil use per capita^c^
industrialized					
Hungary	Budapest	1.3	5.1	7.9	2.7
Italy	Genoa	1.4	3.2	9.2	3.1
South Korea	Pohang	1.2	2.9	7.4	4.6
small-scale					
Dominica	Gwo Woche/Bwa Mwego	2.0	21.0	6.0	0.6
India	Chang Naga and Khasi	2.4	44.0	3.9	0.5
Indonesia	Karo Batak	2.6	32.0	2.9	0.8

^a^Total fertility rate (TFR) and infant mortality rate (IMR) (Population Reference Bureau 2013).

^b^Healthcare expenditure as % of GDP (World Bank 2011).

^c^Oil use *per capita* in 1000s of kg (World Bank 2007).

The caretaking environments of industrialized and small-scale populations cluster into two contrasting categories. As an illustration of this clustering, table 1 presents national-level socio-economic indicators for the focal populations. Oil use *per capita* and health expenditure, for instance, show that small-scale populations tend to exist in relatively less industrialized nations that spend less on public health. Industrialized populations, on the other hand, have lower fertility and infant mortality rates. These are probably conservative estimates of the differences [14], however, as fertility and mortality rates in small-scale populations are often much higher than the national average. Further, there are differences that are not included in table 1, such as the relative importance of infrastructural help for mothers in industrialized populations, and kin networks in small-scale populations. For these reasons, we predict that this clustering will lead to convergent evolution of maternal caretaking heuristics within the clusters. Industrialized/small-scale differences in parental behaviour were suggested previously by LeVine [15,16], and the contrast in industrialized versus small-scale cognitive styles is one of the important ones in Henrich *et al.*'s [17] call for increasing use of comparative studies.

## 2. Material and methods

Third-party judgements of whether a hypothetical mother would engage in a caretaking activity with regards to her hypothetical child were collected using a factorial vignette experiment in seven populations. A total of 32 vignettes, consisting of every possible combination of five binary factors (2^5^=32), was used to describe the mother and child. To minimize respondent fatigue, we presented each with one of eight vignette sets of eight vignettes each (see the electronic supplementary material, table S1), strategically allocating vignettes using the design-of-experiments function in JMP® 8 software [18] so that all main and first-order-interaction effects could be estimated without confounding. The vignette sets and factors were identical to those described in an earlier study [3]. In essence, there were four unique sets of vignettes each used twice to counterbalance the ordering of vignette presentation. So, the vignettes in set A and E, for instance, are identical but presented in different order.

The vignettes read as follows:

There's a woman from a nearby village in her [*mid-20s/late 30s*]. She is [*incapable/capable*] of providing basic food goods to her family compared to the average person in her village or community. She has a little [*boy/girl*] who is [*3 months/2 years*] old. The child is [*often/rarely*] sick.

Respondents from each population were chosen using a random sampling scheme stratified by age, yielding an approximately equal number of respondents from the age categories: 18–25, 26–35, 36–45 and 46+ years old. The presentation of each vignette was followed by four caretaking scenarios of varying severity—i.e. survival stakes—for the mother and her offspring presented in randomized orderings. Karo Batak was an exception, as the severity component presented to that population only included variation in severity for the child, but only the low-severity wordings for the mother. The wordings of the severity component are spelled out in the electronic supplementary material, table S2. In each, the respondent was asked how likely the hypothetical mother would be to engage in the caretaking behaviour based on her ‘observation of other women in the same or similar situations’ on a five-point scale: very likely (+2), likely (+1), neutral (0), unlikely (−1) and very unlikely (−2). Words but not values were presented to respondents. The wording of questions was used so that women would report what they have seen women in their community do, rather than what they thought they themselves were supposed to do. Previous work [3] suggests that the protocol is effective in this regard. All materials were translated to the appropriate languages for administering in each of the seven populations.

We used least-squares linear regression models estimated in Stata® 12 with robust standard errors to adjust for multiple judgements from each respondent [19]. Separate models were built for the industrialized and small-scale samples, and for each population by itself. Each model included terms for: severity for mother (0=less,1=more); severity for child (0=less,1=more); hypothetical mother's age (0=mid−20s,1=late 30s) and access to resources (0=food insecure,1=food secure); child's age (0=three months,1=2years), sex (0=boy,1=girl) and viability (0=often sick,1=rarely sick); interactions of theoretical interest that found support in a previous study [3]: resource access and child's sex, mother's age and child's sex, and mother's age and child's viability; dummy variables for vignette sets (*n*=7); and, respondent age in years. The industrialized and small-scale models also included dummy variables for populations (*n*=6). Justifications for the contrasts used (levels for each factor) and the specific predictions based on how each factor impinges on the fitness costs and benefits of care (not tested here) have been detailed in a previous publication [3].

To statistically compare effects in industrialized versus small-scale populations, cross-model comparisons were carried out using a version of the Chow test [20] for regressions with robust standard errors. This is a two-sided Wald *χ*^2^ test of the null hypothesis that the coefficient for a variable in one model minus the coefficient for that variable in another model equals zero. Statistical significance in these analyses was set at *α*=0.05. To assess whether variation in caretaking environments were behind these differences, we regressed the coefficients from the population-specific models on the national-level statistics presented in table 1. We used linear models in all cases, except for resource access as those estimates fit better to quadratic models. Because of the small number of cases, we assessed fit using adjusted *R*^2^.

## 3. Results

We collected 8128 judgements from 274 respondents (table 2). The data have been made publicly available on the data archiving site figshare [21].

**Table 2.** Descriptive statistics for the study populations.

	sample size		respondent age (years)	
	judgements	respondents	*M*	s.d.
industrialized				
Budapest	1184	37	39.5	15.9
Genoa	1440	45	36.6	14.2
Pohang	544	17	31.3	8.3
combined	*3168*	*99*	*36*.*8*	*14*.*2*
small-scale				
Bwa Mwego/Gwo Woche	1184	37	40.3	16.0
Chang Naga	1536	48	35.3	10.2
Khasi	1600	50	29.9	7.7
Karo Batak	640	40	34.5	11.4
combined	*4960*	*175*	*34*.*7*	*11*.*8*
total	8128	274	35.4	12.8

### 3.1 Cross-model comparisons

**Figure 1.**
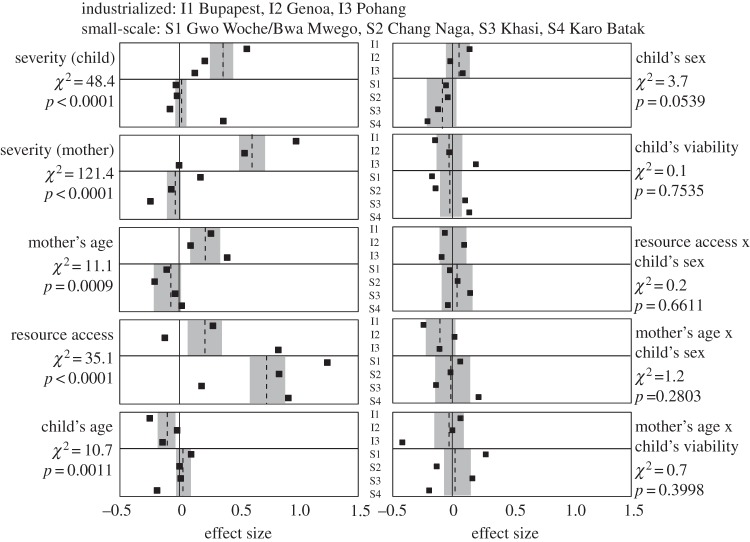
Effects estimates from the industrialized, small-scale and population-specific models: hashed lines and grey areas are point estimates and 95% CIs from the industrialized (upper) and small-scale (lower) models; black squares are point estimates for the population-specific models. **χ**^2^ and *p*-values are Chow tests for the equality of effect estimates in the industrialized and small-scale models.

For the first set of analyses, we estimated one model using all of the data from the industrialized sample and one model using all of the data from the small-scale sample, controlling for population (and other) effects, and then conducted cross-model comparisons of coefficients. The model coefficients and cross-model comparisons are illustrated in figure 1 as hashed lines with grey confidence intervals, and spelled out in detail in the electronic supplementary material, table S3. In summary, and as predicted, three out of five main effects, plus both severity effects, showed statistically significant industrialized/small-scale differences. One of the other main effects, child's sex, was nearly significant. The fifth main effect, child's viability, and all of the interaction effects showed no cross-model difference, and none of these factors exerted more than a negligible effect on judgements in either sample.

In the industrialized populations, there were positive effects of both severity factors and mother's age, but none to negative-but-negligible effects in the small-scale populations. A severe scenario for the child led to a one-third of a step increase in judgements from the industrialized sample, but had no effect in the small-scale sample. A severe scenario for the mother led to almost a two-thirds of a step increase in judgements from the industrialized sample, but only a negligible effect in the small-scale sample. Respondents in the industrialized sample judged the hypothetical woman in her late thirties around 21% of a step more likely to provide care than the hypothetical woman in her mid-twenties. The effect of mother's age in the small-scale sample was negative but negligible.

Child's age and sex had opposite, but relatively small, effects in the industrialized versus small-scale samples. Respondents in the industrialized sample judged the hypothetical woman 12% of a step less likely to provide care to a 2 year old compared with a three-month old. The effect of child's age in the small-scale sample was negligible. When the hypothetical child was female, there was a negligible increase in judgements in the industrialized sample, and a negligible increase in the small-scale sample.

Access to resources, as measured by food security, had positive effects on judgements in both samples, but the effect was almost three-and-one-half times as large in the small-scale populations. A hypothetical food-secure woman was judged as 20% of a step more likely to provide care in the industrialized sample, and almost 75% of a step more likely in the small-scale sample. To look at this another way, consider overall accounting for main and severity effects in the industrialized sample. Together, moving from hypothetical mothers who are older and have insecure food resources, who are making a care decision about children who are younger, female and often sick, and who are faced with a high-severity care situation for both herself and her child, to mothers with the opposite conditions, we predict a 125% of a step increase in the probability of providing care. Further, only 16% of that change is attributable to resource access. That contrasts with the small-scale populations where it predicts an increase of just 54%, and resource access accounts for all of that change (and more, actually, as a number of the effects are negative).

### 3.2 Estimated coefficients by national-level indicators

**Figure 2.**
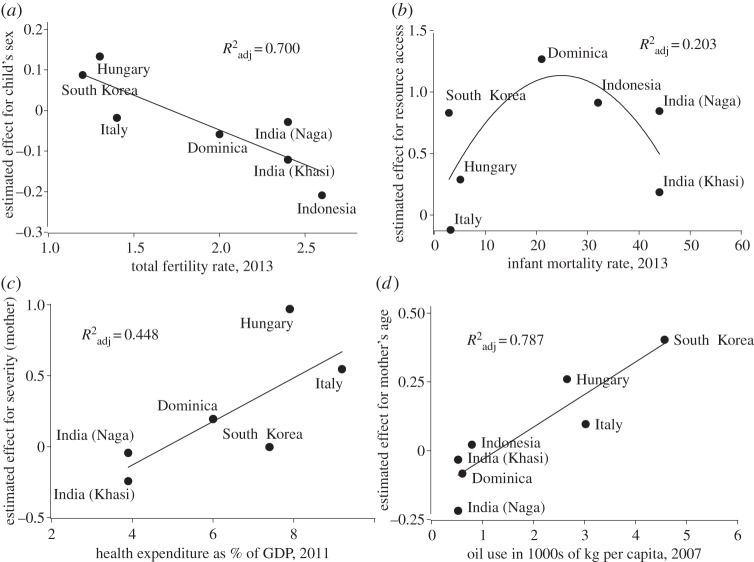
Examples of the relationship between population-specific effects of binary factors in a vignette experiment designed to measure maternal care heuristics and national-level socio-economic indicators: (*a*) effect of offspring sex (female) by fertility rate; (*b*) effect of having secure access to resources by infant mortality rate; (*c*) effect of severity of the scenario to the mother by health expenditure as per cent of GDP, and (*d*) effect of a relatively older mother by oil use *per capita*. All effects are incremental changes in ratings on a five-point scale ranging from very unlikely (−2) to very likely (+2) to provide care.

There was considerable variation in responses by population (see the electronic supplementary material, table S4). Effect estimates from the population-specific models (as shown in the electronic supplementary material, table S5, and by the black squares in figure 1) were regressed on the national-level socio-economic indicators presented in table 1. In all, total fertility rate, infant mortality rate, health expenditure and oil use were reasonable predictors of substantial portions of the variation in effect estimates. One-third of the regressions (eight of 24) had adjusted *R*^2^ values of 0.350 or greater (see the electronic supplementary material, table S6). Figure 2 plots one example for each socio-economic factor: (*a*) effect of child's sex by total fertility rate; (*b*) effect of resource access by infant mortality rate; (*c*) effect of severity (mother) by health expenditure *per capita*; and (*d*) effect of mother's age by oil use.

## 4. Discussion

Our study used a factorial vignette experiment to elicit judgements from women about the probable maternal care decisions of a hypothetical woman and her child. The research design allowed us to: (i) leverage the collective knowledge of women in seven disparate populations about maternal care heuristics used in those societies; (ii) estimate the effects of a handful of fitness-relevant factors and their interactions without confounding; and (iii) test for the evolutionary logic of the maternal care decision rules. We found that, on average, women in industrialized and small-scale societies are responsive to a different subset of fitness-relevant factors. We also found a graded relationship between quantitative aspects of the heuristics and adaptively relevant aspects of the caretaking environments. Taken together, these findings suggest that the contrasts in caretaking heuristics between the two types of populations may be the result of convergent evolution within the two clusters.

As an example of an adaptive difference between the two types of population, mothers in the industrialized sample gave higher judgements on average in response to the scenario's severity for herself and her child, and lower judgements for older children; the effect of these factors in the small-scale sample were negligible. This is consonant with the development of maternal numbness in environments with high infant and child mortality referred to by Scheper–Hughes [22] as ‘death without weeping’. Pay-offs may be too small to support nuanced modulation to these factors in small-scale societies owing to high levels of care-independent (extrinsic) infant and child mortality rates [23,24]. The findings contradict LeVine's [16] hypothesis that mothers in urban-industrial populations should favour toddlers who are more primed for inculcation with traits that increase their competitiveness in those environments, while mothers in other types of populations should favour infants (i.e. 1 to 2 year olds) whose survival depends on that care.

In addition, older mothers were more likely to provide care in industrialized societies; there was no evidence of an age effect in the small-scale sample. The likely explanation is that younger women in industrialized societies are likely to allocate larger proportions of their energy and resources towards the accumulation of embodied capital that can be used to invest in children at a later age [25]. Finally, mothers in both samples modulated their care based on access to resources, as measured by food security. The hypersensitivity of mothers in the small-scale sample may reflect the power of this factor to override all else with regards to reproductive strategies in resource-precarious environments [26], and echoes the conclusion by one of the authors that access to resources is the most important factor shaping maternal care heuristics in a small-scale population [3]. It is also consonant with the primacy of resource access in shaping maternal care heuristics in birds [10], a similarity that might be attributable to high infant mortality and the lack of an institutionalized safety net to assist when resources are scarce.

The between-cluster differences should not be interpreted as a lack of within-cluster variation. Figure 1 illustrates both a comparison of heuristics on average in the industrialized and small-scale samples, and the population-specific heuristics. Taken individually, the populations here clearly exhibit idiosyncratic elements in their maternal care heuristics. A previous analysis of the Karo Batak data [3], for example, suggested that their heuristics included some culturally specific nuances (e.g. significant interaction effects) that were consonant with parental care strategies documented elsewhere using quantitative ethnographic methods [27,28]. In this study, none of the interaction effects displayed a significant industrialized/small-scale difference. Regardless, the differences that were found in other factors while controlling for population effects have clear adaptive significance, as discussed above, suggesting convergent evolution of caretaking behaviour in similar environments [11]. Despite recent work demonstrating a genetic underpinning to human maternal behaviour [29–33], without evidence for interpopulation variation in candidate genes, it would be prudent to look for a non-genetic cause for the differences. We suggest they reflect either facultative responses within existing behavioural reaction norms [7,34] or the parallel products of transmitted culture [35].

This study supports the idea that industrialization (or, more importantly, the changes in important aspects of caretaking environments that occurred with industrialization) may have been as important a ‘watershed’ in the evolution of parental strategies as the shift from ‘low density’ (forager and horticulturalist) to ‘high density’ (agricultural and industrial) society [36]. These changes in the caretaking environment, such as shifts from kin- to institutionalized support and increases in expenditures on public health, may drive changes in maternal caretaking strategies. The regressions of estimated effects on national-level socio-economic indicators (see figure 2 and the electronic supplementary material, table S6) show dose-responses (aka gradients) that strongly support this contention. Although they contradict one of his specific hypotheses (e.g. about offspring-age-specific strategies), the results of this study support LeVine's [15,16] contention that urban-industrial societies adopt qualitatively different parental strategies than other type of societies.

Our approach [13] allowed us to estimate the effect of various factors on the decision-making process and, in doing so, we assumed that the process was an additive one. There are analogues of this decision-making process in the legal arena where judges have been shown to make decisions by increasing or decreasing sentences incrementally based on the presence or absence of various factors [9,37]. We hypothesize that similar heuristics should be observed in other populations. More specifically, we predict that, on average, the subset of fitness-relevant factors used in the maternal decision-making process will differ in industrialized and small-scale societies. The former tending to modulate maternal behaviour on the perceived severity of the situation for herself and her child, and her own reproductive value and access to resources; the latter tending to modulate behaviour strongly on resource access.

## Data accessibility

Data from this study publicly available at: http://dx.doi.org/10.6084/m9.figshare.1172115.
